# Effects of the methanol root extract of *Carpolobia lutea* on sperm indices, acrosome reaction, and sperm DNA integrity in cadmium-induced reproductive toxicity in male Wistar rats

**DOI:** 10.5935/1518-0557.20200036

**Published:** 2020

**Authors:** Adeniran Oluwadamilare Akinola, Adekunle Wahab Oyeyemi, Oluyemi O Daramola, Yinusa Raji

**Affiliations:** 1 Department of Physiology, University of Medical Sciences, Ondo City, Ondo State, Nigeria; 2 Department of Physiology, Igbinedion University Okada, Edo State, Nigeria; 3 Laboratory for Reproductive Physiology and Developmental Programming, Department of Physiology, University of Ibadan, Ibadan, Oyo State, Nigeria

**Keywords:** *Carpolobia lutea* root extract, cadmium, sperm analysis, acrosome reaction, sperm DNA

## Abstract

**Objective:**

Oxidative stress is a mechanism of cadmium-induced reproductive dysfunction. *Carpolobia lutea* is a free radical scavenger. Our study investigated the potential protective effects of *Carpolobia lutea* root methanol extract against cadmium-induced reproductive toxicity.

**Methods:**

We obtained the *Carpolobia lutea* root in Akure, and it was authenticated at the Forestry Research Institute of Nigeria (FRIN) herbarium, Ibadan, Nigeria, with FHI number 109784. We used Soxhlet extraction to obtain its methanol extract. We used thirty male Wistar rats (150-170g) in this study, (n=5 per group), and treated them as follows: Control (1 ml/kg normal saline), Cd (2 mg/kg), Cd+MCL (2 mg/kg+100 mg/kg), Cd+MCL (2 mg/kg+200 mg/kg), MCL (100 mg/kg), MCL (200 mg/kg). We administered *Carpolobia lutea* orally for 8 weeks. We administered a single dose of 2 mg/kg of cadmium intraperitoneally. We assessed the sperm profile using a computer-aided sperm analyzer. Under microscopy, we determined the sperm acrosome reaction and the DNA damage. We measured the seminal fructose level using spectrophotometry, and the data were analyzed using ANOVA at *p*<0.05.

**Results:**

Cd+MCL (2mg/kg+200 mg/kg) significantly increased sperm count (339.0±25.0 *vs.* 29.0±4.5 million/mL), motility (80.0±0.2 *vs.* 55.0±4.9%), viability (68.7±2.7 *vs.* 31.3±2.9%) and decreased abnormal sperm (28.3±1.7 *vs.* 43.3±2.5%), relative to the cadmium group. Cd+MCL (2mg/kg+200 mg/kg) significantly increased acrosome reaction (68.0±7.5 *vs.* 15.2±2.4%) and seminal fructose level (0.49±0.06 *vs.* 0.28±0.06 mmol/L) relative to the cadmium group. Cd+MCL (2mg/kg+200 mg/kg) significantly decreased sperm DNA damage (14.1±1.6 *vs.* 35.9±5.3%) in relation to the cadmium group.

**Conclusions:**

*Carpolobia lutea* root extract improves the sperm variables of rats exposed to cadmium.

## INTRODUCTION

Infertility affects approximately 15% of all couples trying to conceive. Male infertility is the sole or a contributing factor in roughly half of these cases, and no identifiable cause can be found in over 25% of infertile males (Yeşilli *et al.,* 2005). However, the etiology of the male factor infertility is poorly understood, while some individuals may be genetically predisposed to be sub-fertile (Reijo *et al.,* 1996). There are major epigenetic factors implicated as potential causes of male infertility. The most common idiopathic oligoasthenoteratozoospermia (OAT) (Hirsch, 2003), which is a condition in which sperm concentration, the proportion of morphologically normal sperm and the proportion of motile sperm, are all lower than the World Health Organization reference values (Cooper *et al.,* 2010). Despite extensive research, a successful treatment for OAT has not yet been developed. Many recent studies have focused on oxidative stress and its possible role in the pathogenesis of male infertility. In physiological conditions, spermatozoa produce small amounts of reactive oxygen species (ROS), and various scavengers act to reduce the concentration of these ROS in the seminal plasma. However, excessive production and/or reduced clearance leads to oxidative stress within the sperm, resulting in reduced motility (Kao *et al.,* 2008) and defective membrane integrity (Agarwal *et al.,* 2003). One of the reactive oxygen species inducers is environmental exposure to toxicants.

Cadmium (Cd) is a heavy metal and a relevant environmental toxicant. The general population is exposed to cadmium through contaminants found in drinking water and food. In addition, occupational exposure to cadmium occurs during mining and the production of batteries and pigments that contain cadmium. Industrial activities, e.g. smelting and refining of metals, and municipal waste incineration release cadmium into the atmosphere (Siu *et al.,* 2009). Tobacco smoke is another source of cadmium exposure (Blanco *et al.,* 2007). Acute cadmium chloride exposure causes significant reproductive damage through increased oxidative stress, histological alteration, (necrosis, edema etc.) and spermatological damage (decreased sperm motility and sperm concentration, and increased abnormal sperm cells). Cadmium toxicity is associated with severe damage to various organs, particularly the testes, in both humans and animals (Fouad *et al.,* 2009). Cadmium impairs the reproductive capacity by causing severe testicular degeneration, seminiferous tubule damage and necrosis in rats (Burukoğlu & Bayçu, 2008).

Many natural herbal and nutritional aphrodisiacs enhance sexual drive and pleasure in both men and women. Studies have validated that some herbs have aphrodisiac activity (Rajeshwar *et al.,* 2005). *Carpolobia lutea* G Don (Polygalaceae) is one of natural herb and nutritional aphrodisiac. *It* is a shrub or small tree of up to 5cm-high. It is widely found in tropical Africam, where it is known as ikpafum in Ibibio; Abekpok ibuhu in Eket; Angalagala in Ibo; Egbo oshunshun in Yoruba and cattle stick in English. It is used to facilitate delivery and treat male sexual disorders because of its aphrodisiac effect (Mitaine-Offer *et al*., 2002). An ethno-botanically decoction of the root is used by Ibibio’s of Akwa Ibom state of Nigeria as an aphrodisiac (Ajibesin *et al.,* 2008), and malarial remedy. It has analgesic and androgenic properties, reputed to cure rheumatism, insanity, fever, skin infection, vermifuge, venereal diseases, combat sterility and promote childbirth (Muanya & Odukoya, 2008). *Carpolobia lutea* leaves are used for the treatment of ulcers and diarrhea (Nwafor & Bassey, 2007), as well as malarial remedy in some part of Nigeria. The root can also be used as an anti-inflammatory and anti-arthritic agent (Iwu & Anyanwu, 1982), vermifuge, facilitate childbirth and to treat sterility and headache (Mitaine-Offer *et al.,* 2002).

The present study evaluated the *Carpolobia lutea* root methanol extract effect on epididymal sperm parameters, sperm capacitation and acrosome reaction, seminal fructose level and sperm chromatin integrity in cadmium-treated male Wistar rats.

## MATERIALS AND METHODS

### Chemical

The cadmium chloride salt came from Loba chemie, PVT, India.

### Plant harvest and extraction

The *Carpolobia lutea* root was obtained from Ijare, a village *via* Akure, in the Ondo state. The plant was authenticated at the Forestry Research Institute of Nigeria (FRIN) herbarium, in Ibadan, Nigeria, with FHI number 109784. The *Carpolobia lutea* root was air dried and pulverized. The pulverized *Carpolobia lutea* root (5.20kg) was subjected to Soxhlet extraction using pure methanol as the solvent. Methanol containing the extract was then filtered and the solvent was vacuum-distilled at 4ºC in a rotary evaporator. The remaining extract was finally dried in a vacuum oven at 30ºC for 2 hours to ensure the removal of any residual solvent. The powdery mass yielded 87.88g (1.69% yields), which was then stored for the study. The extract’s fresh solution was prepared in distilled water.

### Phytochemical screening

The *C. lutea* root methanol extract phytochemical screening determined the presence of chemical constituents such as flavonoids, simple sugar, alkaloids, tannins, saponins, phlobatannins, cardiac glycosides and anthraquinones, following the method of Odebiyi & Sofowora (1978) and Trease & Evans (1989).

### Acute Toxicity Study

The whole animal acute toxicity study was carried out according to the Organization for Economic Cooperation and Development (OECD) Limit test guidelines (2001). Nine male rats were used for the study. They were divided into 3 groups of 3 rats each. The animals were fasted overnight (no food, but they were given water). Groups 1, 2 and 3 received a single oral dose of 1000 mg/kg, 2000 mg/kg and 5000 mg/kg of *C. lutea* root extract, respectively. We observed the animals for 2 hours for any behavioral and neurological features, then intermittently over the next 72 hours and daily for 14 days, with special attention to any moribound state or death.

### Animals and Experimental Design

We used Wistar adult male rats (150 to 170 gram) housed in well-ventilated rat cages in the Central Animal House, College of Medicine, University of Ibadan for this study. We kept them under standard laboratory conditions of 12-hour light and 12-hour dark cycle and were fed with standard commercial rat pellets (Ladokun feeds Limited, Ibadan, Nigeria), and allowed access to water *ad libitum*. We acclimatized them for two weeks. We weighed the animals weekly throughout the study. For this study, we followed the guiding principles for research involving experimental animals, as recommended by the Declaration of Helsinki, as well as the Guiding principles for the use and care of animals (World Medical Association & American Physiological Society, 2002). We randomly divided the animals into six groups, with five animals per group and treated as follows:

Group 1: Control (1.0 ml/kg normal saline, vehicle);
Group 2: Cd (2 mg/kg);
Group 3: Cd+MCL (2 mg/kg+100 mg/kg);
Group 4: Cd+MCL (2 mg/kg+200 mg/kg);
Group 5: MCL (100 mg/kg);
Group 6: MCL (200 mg/kg).

We treated the animals with vehicle and methanol extract of *Carpolobia lutea* root, orally for 8 weeks and cadmium (single dose intraperitoneally). Twenty-four hours after the last administration, we anesthetized the animals with 50 mg/kg of sodium thiopentone before they were sacrificed. The animals’ testes and epididymis were harvested and used for sperm analysis, sperm capacitation and acrosome reaction, seminal fructose level and sperm chromatin integrity assessments.

### Sperm Analysis

We studied sperm concentration, sperm kinetics and motility using the Computer assisted sperm analyzer (CASA) JH-6004 - Sperm Quality Analyzer. The sperm viability study (percentage of live spermatozoa) was assessed by microscopy, using Raji *et al*.’s method (2003). We studied sperm morphology by microscopy according to Sarkar *et al.* (2006).

### Sperm Capacitation and Acrosome Reaction

This was assessed by microscopy according to the methods described by Toyoda & Chang (1974) and Feng *et al*. (2007).

### We assessed sperm DNA damage using aniline blue staining techniques

We used microscopy using the methods described by Wong *et al*. (2008) and Park *et al*. (2011).

### Seminal fructose level

We ran this analysis using spectrophotometry, following the method described by Zahoor *et al*. (2010).

### Statistical analysis

We expressed our results as mean ± SEM for five animals per group. We used one-way variance analysis (ANOVA) to assess the statistical significance of the data. We used Fisher΄s Least Significant Difference (LSD) test for post hoc analysis (Multiple comparison). *P*<0.05 was considered significant.

## RESULTS

### Acute Toxicity Study

Table 1 shows the acute toxicity of *C. lutea* root. The male Wistar rats given *C. lutea* up to 5,000 mg/kg neither died nor displayed any signs of toxicity after a 14-day observation.

**Table 1:** *C. lutea* root extract acute toxicity using male Wistar rats

Treatment Group	Survival (%)	Death (%)
MCL (1000 mg/kg)	100	Zero
MCL (2000 mg/kg)	100	Zero
MCL (5000 mg/kg)	100	Zero

### Phytochemical Screening

Table 2 shows the phytochemical screening of methanol extract of *C. lutea* root. It shows that flavonoids, saponins, anthraquinones, alkaloids, tannins, cardiac glycosides, terpenes and simple sugar were present the in the *C. lutea* root methanol extract.

**Table 2:** Phytochemical screening of *C. lutea* root methanol extract

Secondary Metabolites	Results
Alkaloids	**++**
Tannins	**++**
Saponins	**+++**
Anthraquinones	**+**
Flavonoids	**+**
Glycosides	**-**
Cardiac glycosides	**++**
Terpenes	**+**
Simple sugars	**++**

**+ Present - Absent**

### Sperm variables

#### Sperm Count

**Figure 1:**
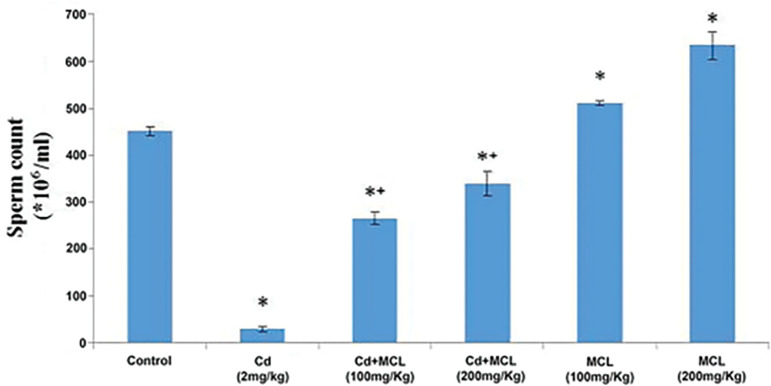
Effect of *C. lutea* root methanol extract on sperm count of cadmium-induced reproductive toxicity in male Wistar rats. Values are expressed as Mean±SEM, n=5. Cd-Cadmium, MCL-methanol extract of *C. lutea*. ^*^,^+^ *p*<0.05 was considered significant when compared with control and cadmium groups, respectively.

Figure 1 shows that sperm count was significantly decreased in the Cd (2 mg/kg), Cd+MCL (100mg/kg) and Cd+MCL (200mg/kg) groups when compared with the control group. The sperm count was significantly increased in MCL (100mg/kg) and MCL (200mg/kg)-treated groups when compared with the Control Group. In addition, there was significant increase in the MCL (100mg/kg) and MCL (200mg/kg) Groups when compared with the Cd (2mg/kg) Group.

#### Sperm Viability

**Figure 2:**
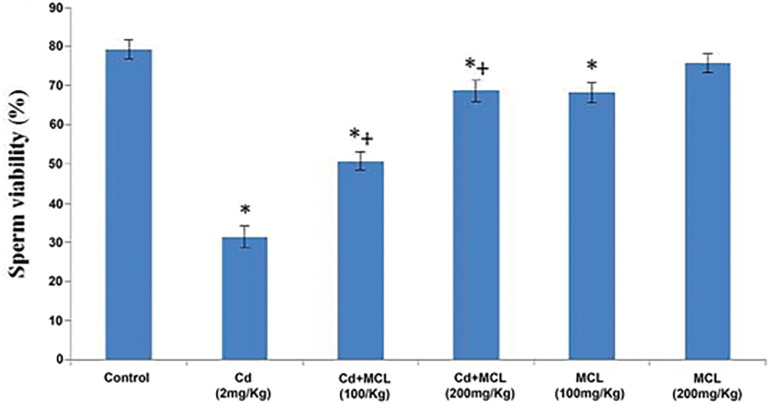
Effect of *C. lutea* root methanol extract on sperm viability of cadmium-induced reproductive toxicity in male Wistar rats Values are expressed as Mean±SEM, n=5. Cd-Cadmium, MCL-methanol extract of *C. lutea*. ^*^,^+^ *p*<0.05 were considered significant when compared with control and cadmium groups, respectively.

Figure 2 shows that sperm viability was significantly decreased in the Cd (2mg/kg), Cd+MCL (100mg/kg), Cd+MCL (200mg/kg) and MCL (100mg/kg)-treated groups, when compared with the Control Group, while there was a significant increase in the Cd+MCL (100mg/kg) and Cd+MCL (200mg/kg)-treated groups when compared with the Cd (2mg/kg)-treated group.

#### Sperm Abnormality

**Figure 3:**
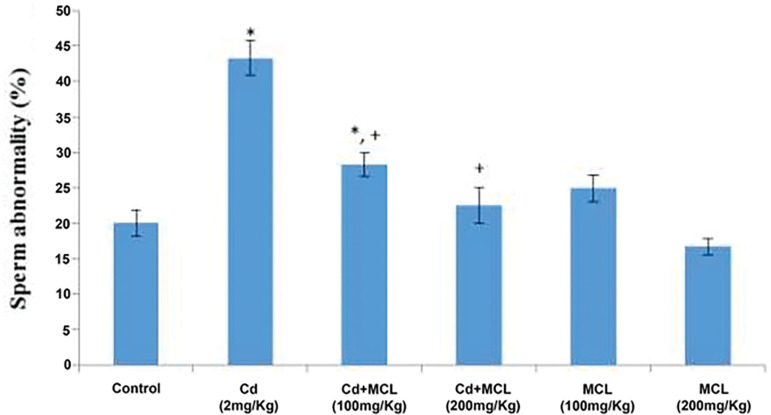
Effect of *C. lutea* root methanol extract on sperm abnormality of cadmium-induced reproductive toxicity in male Wistar rats. Values are expressed as Mean±SEM, n=5. Cd-Cadmium, MCL-methanol extract of C. lutea. ^*^,^+^ *p*<0.05 were considered significant when compared with control and cadmium groups, respectively.

Figure 3 shows that sperm abnormality was significantly increased in the Cd (2mg/kg) and Cd+MCL (100mg/kg)-treated groups, when compared with the Control Group. There was a significant decrease in sperm abnormality in the Cd+MCL (100mg/kg) group, when compared with the Cd (2mg/kg) group.

#### Sperm Motility

**Figure 4:**
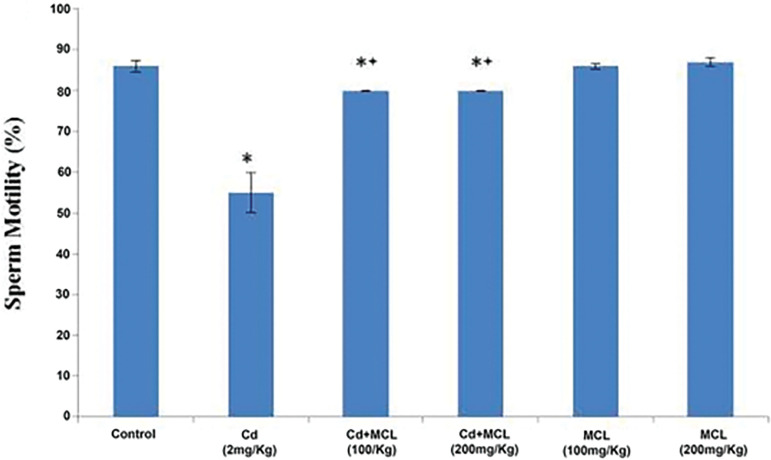
Effect of *C. lutea* root methanol extract on sperm motility of cadmium-induced reproductive toxicity in male Wistar rats Values are expressed as Mean±SEM, n=5. Cd-Cadmium, MCL-methanol extract of C. lutea. ^*^,^+^ *p*<0.05 were considered significant when compared with control and cadmium groups, respectively.

Figure 4 shows that sperm motility was significantly decreased in the Cd (2mg/kg), Cd+MCL (100mg/kg) and Cd+MCL (200mg/kg)-treated groups, when compared with Control animals. In addition, there was a significant increase in sperm motility in the Cd+MCL (100mg/kg) and MCL (200mg/kg) groups when compared with the Cd (2mg/kg)-treated group.

#### Total Sperm Detected

Table 3 shows that the total sperm detected was significantly decreased in the Cd (2mg/kg), Cd+MCL (100mg/kg) and Cd+MCL (200mg/kg) -treated groups, when compared with the control animals. It was significantly increased in the MCL (100mg/kg) and MCL (200mg/kg) -treated groups, when compared with the Control group. Furthermore, there was a significant increase in the Cd+MCL (100mg/kg) and MCL (200mg/kg) -treated groups, when compared with the Cd (2mg/kg)-treated group.

**Table 3:** Effects of *Carpolobia lutea* root Methanol Extract on total sperm detected, total motile sperm, progressive motility, non-progressive motility and immotile sperm in Male Wistar Rats Exposed to Cadmium

S/N	Groups	Total Sperm Detected	Total motile Sperm	Progressive Motility (%)	Non Progressive Motility (%)	Immotile sperm (%)
1	Control	1107±20.7	955±30	41±0.76	46±0.60	14±1.26
2	Cd (2mg/kg)	38±2.6*	25±16*	7±0.82*	48±4.06	45±4.88*
3	Cd+MCL (100mg/Kg)	614±64.2*^+^	484±16*^+^	33±0.75*^+^	48±0.69	20±0.06*^+^
4	Cd+MCL (200mg/Kg)	835±61.5*^+^	667±50*^+^	37±0.64*^+^	43±0.84	20±0.22*^+^
5	MCL (100mg/Kg)	1256±11.8*	1080±14*	39±1.75	47±2.17	14±0.66
6	MCL (200mg/Kg)	1557±73.1*	1384±79*	40±0.95	48±0.17	11±1.11

Values expressed in mean ± SEM,

^*,+^*p*<0.05 show a significant difference when compared with Control and Cd, respectively.

#### Total Motile Sperm

Table 3 shows that the total motile sperm count was significantly decreased in Cd (2mg/kg), Cd+MCL (100 mg/kg) and Cd+MCL (200mg/kg) -treated groups when compared with the control animals, while significant increases were seen in MCL (100mg/kg) and MCL (200mg/kg) groups when compared with the control group. In addition, total motile sperm was significantly increased in Cd+MCL (100mg/kg) and MCL (200mg/kg) groups when compared with the Cd (2 mg/kg) group.

#### Progressive, Non-progressive and Immotile Sperm

Table 3 shows that sperm progressive motility was significantly decreased in the Cd (2mg/kg), Cd+MCL (100mg/kg) and Cd+MCL (200mg/kg) groups when compared with the control group; while significant increases were seen in the Cd+MCL (100mg/kg) and Cd+MCL (200mg/kg) when compared with Cd (2 mg/kg) group. Immotile sperm was significantly increased in the Cd (2 mg/kg), Cd+MCL (100 g/kg) and MCL (200 mg/kg) groups, when compared with the control group, while significant decreases were seen in the Cd+MCL (100mg/kg) and MCL (200mg/kg) groups when compared with the Cd (2mg/kg) group.

#### Sperm Velocity

Table 4 shows that the sperm average path velocity was significantly decreased in the Cd (2mg/kg), Cd+MCL (100mg/kg) and Cd+MCL (200mg/kg) groups when compared with the control group, while it was significantly increased in the MCL (100mg/kg) and MCL (200mg/kg) treated groups when compared with the control group. The sperm average path velocity was significantly increased in the Cd+MCL (100mg/kg) and Cd+MCL (200mg/kg) groups, when compared with the Cd (2mg/kg) group.

**Table 4:** Effects of *Carpolobia lutea* root m ethanol extract on sperm kinetics in Male Wistar Rats Exposed to Cadmium

S/N	Groups	Average path (µm/s)	Curvilinear (µm/s)	Straight line (µm/s)	Amplitude of lateral head (µm/s)	Beat Cross Frequency (Hz)	Line Moving (%)
1	Control	16.6±0.26	27.8±0.50	7.4±0.11	0.89±0.018	3.32±0.096	29.4±0.429
2	Cd (2mg/kg)	3.4±0.52*	4.2±0.56*	2.5±0.35*	0.19±0.025*	0.46 ±0.028*	7.1±0.817*
3	Cd+MCL(100mg/Kg)	14.3 ±0.03*^+^	21.4±0.22*^+^	6.3±0.03*^+^	0.74 ±0.004*^+^	2.39 ±0.024*^+^	28.6±0.142^+^
4	Cd+MCL(200mg/Kg)	15.3 ±0.24*^+^	24.0 ±0.42*^+^	6.9±0.16*^+^	0.80±0.011*^+^	2.67 ±0.054*^+^	30.5 ±1.097+
5	MCL(100mg/Kg)	17.3±0.24*	29.1±0.82	7.4±0.16	0.92±0.020	3.63±0.116*	27.2 ±0.676*
6	MCL(200mg/Kg)	17.6±0.04*	31.5±0.25*	7.5±0.11	0.95 ±0.002*	4.01 ±0.062*	25.8 ±0.542*

Values expressed in mean ± SEM,

^*,+^*p*<0.05 show a significant difference when compared with Control and Cd, respectively.

The sperm curvilinear velocity was significantly decreased in the Cd (2mg/kg), Cd+MCL (100mg/kg) and MCL (200mg/kg) groups, when compared with the Control group, while it was significantly increased in the MCL (200mg/kg) group, when compared with control animals. There was a significant increase in sperm curvilinear velocity of the Cd+MCL (100mg/kg) and MCL (200mg/kg) groups when compared with the Cd (2mg/kg) group.

The straight line velocity was significantly decreased in the Cd (2mg/kg), Cd+MCL (100mg/kg) and Cd+MCL (200mg/kg) groups when compared with control animals, while significant increases were seen them in Cd+MCL (100mg/kg) and Cd+MCL (200mg/kg) -treated groups when compared with the Cd (2mg/kg) group.

### Lateral Sperm Head Amplitude

Table 4 shows that the lateral sperm head amplitude was significantly decreased in the Cd (2mg/kg), Cd+MCL (100mg/kg) and Cd+MCL (200mg/kg) -treated groups, respectively when compared with the control group. In addition, there was a significant increase in the MCL (200mg/kg) -treated group when compared with the control group. Alternatively, there was a significant increase in the MCL (100mg/kg) and MCL (200mg/kg) -treated groups respectively, when compared with the Cd (2mg/kg)-treated group.

#### Sperm Beat Cross Frequency

Table 4 shows that the sperm beat cross frequency was significantly decreased in the Cd (2mg/kg), Cd+MCL (100mg/kg) and Cd+MCL (200mg/kg) -treated groups, respectively; when compared with control animals. In addition, there was a significant increase in the MCL (100mg/kg) and MCL (200mg/kg) treated groups when compared with the control groups. Alternatively, there was a significant increase in the Cd+MCL (100mg/kg) and Cd+MCL (200mg/kg) -treated groups, respectively; when compared with the Cd (2mg/kg) Group.

### Sperm Line Moving

Table 4 shows that sperm line moving was significantly decreased in the Cd (2mg/kg), MCL (100mg/kg) and MCL (200mg/kg) *-*treated groups, when compared with the control group. Alternatively, there was a significant increase in the Cd+ MCL (100mg/kg) and Cd+ MCL (200mg/kg) treated groups, respectively; when compared with the Cd (2mg/kg) Group.

#### Sperm Linearity

Table 5 shows that the sperm linearity was significantly increased in the Cd (2mg/kg), Cd+MCL (100mg/kg) and Cd+MCL (200mg/kg) -treated groups, when compared with control animals. In addition, there was a significant decrease in the MCL (100 mg/kg) and MCL (200mg/kg) groups, when compared with control animals. Alternatively, there was a significant decrease in the Cd+MCL (100mg/kg) and Cd+MCL (200mg/kg) treated groups, respectively; when compared with the Cd (2mg/kg) group.

**Table 5:** Effects of *Carpolobia lutea* root Methanol Extract on Sperm kinetics in Male Wistar Rats Exposed to Cadmium

S/N	Groups	Linearity (%)	Straightness (%)	Wobble (%)	Mean Move angle (Degree)
1	Control	26.6±0.079	44.5±0.045	52.9±4.288	9.6±0.228
2	Cd(2mg/kg)	60.8 ±0.163*	70.5 ±0.358*	86.3±0.673*	1.3±0.165*
3	Cd+MCL(100mg/Kg)	29.2±0.148*^+^	43.6±0.138^+^	67.0 ±0.566*^+^	6.9±0.020*^+^
4	Cd+MCL(200mg/Kg)	28.8±0.479*^+^	45.2±0.414^+^	63.8±0.615*^+^	7.3±0.276*^+^
5	MCL(100mg/Kg)	25.2±0.218*	42.5±0.434*	59.4±0.866*	10.6±0.163*
6	MCL(200mg/Kg)	23.7±0.405*	42.4 ±0.567*	56.0±0.430	122.3±0.349*

Values expressed in mean ± SEM,

^*,+^*p*<0.05 show a significant difference when compared with Control and Cd, respectively.

#### Sperm Straightness

Table 5 shows that sperm straightness was significantly increased in the Cd (2mg/kg) group, when compared with the Control group. In addition, there was a significant decrease in the MCL (100mg/kg) and MCL (200mg/kg) groups, when compared with control animals. Alternatively, there was a significant decrease in the Cd+MCL (100mg/kg) and Cd+MCL (200mg/kg) -treated groups, respectively; when compared with the Cd (2mg/kg) group.

#### Sperm Wobble

Table 5 shows that sperm wobble was significantly increased in the Cd (2mg/kg), Cd+100 mg/kg, Cd+MCL (200mg/kg) and MCL (100mg/kg) -treated groups, respectively; when compared with the control group. Alternatively, there was a significant decrease in the Cd+MCL (100mg/kg) and Cd+MCL (200mg/kg) -treated groups, respectively; when compared with the Cd (2mg/kg) group.

#### Sperm Mean Move Angle

Table 5 shows that sperm mean move angle was significantly decreased in the Cd (2mg/kg), Cd+MCL (100mg/kg) and Cd+MCL (200mg/kg) -treated groups, respectively; when compared with the control group. In addition, there was a significant increase in the MCL (100mg/kg) and MCL (200mg/kg) -treated groups, when compared with control animals. Alternatively, there was a significant increase in the Cd+MCL (100mg/kg) and Cd+MCL (200mg/kg) -treated groups, respectively; when compared with the Cd (2mg/kg) group.

#### Sperm Capacitation and Acrosome Reaction

Table 6 shows that in the acrosome intact uncapacitated sperm, there was a significant increase in the Cd (2mg/kg), Cd+MCL (100mg/kg) and Cd+MCL (200mg/kg) -treated groups, when compared with control animals. On the other hand, there was a significant decrease in the Cd+MCL (100mg/kg) and Cd+MCL (200mg/kg) -treated groups, when compared with the Cd (2mg/kg) group. In acrosome-reacted capacitated sperm, there was a significant decrease in the Cd (2mg/kg), Cd+MCL (100mg/kg) and Cd+MCL (200mg/kg) -treated groups, when compared with control animals. On the other hand, the Cd+MCL (100mg/kg) and Cd+MCL (200mg/kg) -treated groups were significantly increased when compared with the Cd (2mg/kg) group.

**Table 6:** Effects of *Carpolobia lutea* root Methanol Extract on Sperm acrosome reaction, sperm DNA integrity and seminal fructose level in Male Wistar Rats Exposed to Cadmium

S/N	Groups	Acrosome intact uncapacitated sperm (%)	Acrosome reacted capacitated sperm (%)	Chromatin condensation (%)	Seminal fructose (mmol/L)
1	Control	8.4±2.66	91.6±2.66	10.4±1.43	0.54±0.026
2	Cd	84.8±2.40*	15.2±2.40*	35.9±5.26*	0.28±0.061*
3	Cd+MCL(100mg/Kg)	67.0±2.55*^+^	33.0±2.55*^+^	23.0±2.22*^+^	0.40±0.022^+^
4	Cd+MCL(200mg/Kg)	32.0±7.52*^+^	68.0±7.52*^+^	14.1±1.57^+^	0.49±0.064^+^
5	MCL(100mg/Kg)	13.8±3.12	86.3±6.25	11.4±1.10	0.57±0.086
6	MCL(200mg/Kg)	12.0±2.42	88.0±5.83	9.6±1.93	0.53±0.076

Values expressed in mean ± SEM,

^*,+^*p*<0.05 show a significant difference when compared with control and Cd animals, respectively.

#### Sperm Chromatin Integrity

Table 6 shows that abnormal sperm chromatin was significantly increased in the Cd (2mg/kg) and Cd+MCL (100mg/kg) -treated groups, when compared with control animals. On the other hand, there was a significant decrease in the Cd+MCL (100mg/kg) and Cd+MCL (200mg/kg) -treated groups when compared with the Cd (2mg/kg) group.

#### Seminal Vesicle Fructose Level

Table 6 shows that the seminal vesicle fructose level was significantly decreased in the Cd (2mg/kg) group when compared with the Control group. On the other hand, there was a significant increase in the Cd+MCL (100mg/kg) and Cd+MCL (200mg/kg) -treated groups when compared with the Cd (2mg/kg) group.

## DISCUSSION

Cadmium typifies a dangerous environmental, occupational and industrial pollutant. Several studies with experimental animals have reported that the generation of reactive oxygen species (ROS) and its interference with the cellular antioxidant system is one of the major mechanisms by which the toxic effect of cadmium is mediated (Sen Gupta *et al.,* 2004).

The present study showed the efficacy of *Carpolobia lutea* root methanol extract in preventing the toxic effects of cadmium on the rats’ spermatozoa. In this study, we investigated the effects of *Carpolobia lutea* root methanol extract on sperm characteristics, sperm capacitation and acrosome reaction, sperm chromatin integrity and seminal fructose level in cadmium-induced reproductive toxicity of male rats.

The study showed that *C. lutea* root is not toxic and it is safe for oral consumption. The *C. lutea* root extract contains important phytochemical compounds such as alkaloids, tannins, saponins, anthraquinones, flavonoids, cardiac glycosides, terpenes and simple sugar that are similar to the report by Yakubu & Jimoh (2015). Flavonoids and tannins are phenolic compounds, and plant phenolics are a major group of compounds that act as primary antioxidants or free radical scavengers (Li *et al.,* 2009; Sim *et al.,* 2010). Similarly, terpenoids act as regulators of metabolism and play a protective role as antioxidants (Soetan, 2008). Saponins are steroids or triterpenoid glycosides, common in a large number of plants and plant products that are important in human and animal nutrition. Several biological effects have been ascribed to saponins, and these include hypolipidemic, hypoglycemic, anticarcinogenic and antioxidant properties (Elekofehinti *et al.,* 2012). In addition, the administration of alkaloid compound was reported to decrease lipid peroxidation in tissues indicating antioxidant-like activity, which alleviates oxidative stress (Al-Fartosy *et al.,* 2013). Metabolism of simple sugars like glucose will lead to the production of pyruvate. Pyruvate is a substrate necessary for the activity and survival of sperm cells (Egbunike *et al.,* 1986). Muanya & Odukoya (2008) also reported that cardiac glycosides and saponins have antioxidant properties.

The present study showed that cadmium significantly reduced sperm motility, viability, count, while abnormal sperm morphology was increased. The observed reduction in sperm motility, viability and count might be due to the damaging effects of cadmium on spermatogenesis. The adverse effects of cadmium on sperm profile could be ascribed to either the reduction in serum testosterone levels or generation of reactive oxygen species (Lafuente *et al.,* 2001; Waisberg *et al.,* 2003). Sperm is highly susceptible to lipid peroxidation (LPO) because of the abundance of unsaturated fatty acids in the sperm plasma membrane and a very small concentration of cytoplasmic antioxidants (Aitken et al., 1993). The high level of LPO can result in oxidative damage to sperm DNA, disrupt membrane functions, impair motility and possibly have a significant effect on spermatozoa development (Aitken *et al.,* 1989). One of the toxicity indicators due to chemicals on the reproductive system is a reduction in the level of testosterone (Yoshida *et al.,* 2002). Testosterone is essential for the maintenance of the structure and function of the male accessory sex glands. Moreover, reduced or lack of this hormone hinders spermatogenic function (Boockfor & Blake, 1997). The present result on sperm profile was in agreement with the findings of El-Missiry & Shalaby (2000) and El-Demerdash *et al*. (2004), who demonstrated that cadmium can induce lipid peroxidation, testicular tissue necrosis and apoptosis in rats. Exposure to cadmium can induce germ cell apoptosis, which may account for the current decline in male fertility (Akinloye *et al.,* 2006). Another study, carried out by Kasinathan *et al*. (1987) showed that cadmium significantly decreased primary and secondary spermatocytes in the seminiferous tubules. Sen Gupta *et al*. (2004) reported that reactive oxygen species are involved in cadmium-induced testicular damage. In addition, cadmium-induced oxidative stress is well established (Stohs *et al.,* 2001; El-Demerdash *et al.,* 2004). Cadmium administration generates reactive oxygen species at a cellular level (Wang *et al.,* 2004; Kusakabe *et al.,* 2008) and is associated to increased lipid peroxidation (Croute *et al.,* 2005); hence, cadmium-induced ROS generation can increase lipid peroxidation, which leads to testicular tissue damage and reduce spermatogenesis. Indeed, a large proportion of infertile men have increased levels of seminal ROS (Pasqualotto *et al.,* 2000). The improvement in sperm quality and quantity by the *C. lutea* extract and palmitic acid might be attributed to attenuation of oxidative damage by cadmium and stimulation from testosterone biosynthesis.

During the past decades, the quality and fertility potential of sperm has decreased dramatically. Sperm motility has a high correlation with fertility and is an early and sensitive endpoint for evaluating its chemical effects on male fertility (Lifeng *et al.,* 2006). The efficacy of computer-assisted sperm analyzer (CASA) has been demonstrated for use with a variety of species in assessing male reproductive quality, as well as the impact of various treatments on sperm motility. Computer-assisted sperm analyzer enables an objective assessment of different cell characteristics: motion, velocity, and morphology (Verstegen *et al.,* 2002). Our results obtained by motion analysis depict a significant decline in the percentage of spermatozoa with progressive motility, and significant decrease in average path velocity (VAP), curvilinear velocity (VCL), straightline velocity (VSL), amplitude of lateral head displacement (ALP), beat cross frequency (BCF) and significant increase in linearity (LIN), straigthness (STR) and wobble in cadmium-treated rats. These observations confirm the positive relationship between cadmium levels and asthenozoospermia, supporting the hypothesis that environmental cadmium exposure may contribute significantly to reduced sperm motility (Xu *et al.,* 2001; Benoff *et al.,* 2009). Moreover, reduction in a motility parameter, such as the BCF, has damaging effects on sperm motility, since they are indicators of sperm vigor (Duty *et al.,* 2004). Important velocity parameters (VSL, VCL and VAP) directly express sperm motion and decline in sperm velocity, percentage of motile sperm, BCF and ALH parameters can adversely affect fertility (Ban *et al.,* 1999; Kato *et al.,* 2001). ALH is calculated from the amplitudes of the lateral deviations of sperm head about the axis of progression (Mukhopadhyay *et al.,* 2010). It is a valuable measurement, as this is one of the parameters affecting the outcome of *in-vitro* fertilization and sperm penetration ability (Verstegen *et al.,* 2002). The *C. lutea* root methanol extract ameliorated cadmium-induced toxicity in sperm kinetics. The action of *C. lutea* root methanol extract might be due to its antioxidant ability to mop up the toxic effects of cadmium in the testes (Salama & El-Bahr, 2007).

To achieve successful fertilization under normal *in-vivo* conditions*,* mammalian spermatozoa must consecutively undergo capacitation and acrosome reaction (Suarez & Pacey, 2006). The extracellular environment plays a prominent role in achieving these complex events that enable spermatozoa to achieve fertilizing ability at the right time and on the right site (Zhou *et al.,* 2008). The coomassie brilliant blue (CBB) staining technique is most convenient and stable in assessing acrosome reaction than other methods (Zhang *et al.,* 2005). The result of the study showed that the intact acrosome uncapacitated sperm was significantly increased in cadmium treated rats. On the other hand, the co-administration of C. lutea root methanol extract with cadmium reduced acrosome intact uncapacitated sperm. The acrosome-reacted capacitated sperm was significantly decreased in the cadmium-treated rats, while significant increase was seen when co-treated with methanol extract of *C. lutea* root. Development of culture systems that allow capacitation and fertilization *in-vitro* has made it possible to determine ions required precisely for capacitation and acrosome reaction. Calcium ion is a prime regulator of sperm motility, capacitation and initiator of acrosome reaction processes (Schuh *et al.,* 2004; Publicover *et al.,* 2007). Also low concentrations of Na^+^ are necessary for sperm capacitation. *C. lutea* root methanol extract may prevent cadmium to replace the metals cofactors from their active site or to bind to a deactivating site of the enzyme itself and disrupt or interrupt activity, which can lead to oxidative stress (Casalino *et al.,* 1997). The extract might also cause the plasma membrane to act as a barrier and also, activate catalytic enzymes (Alvarez & Storey, 1984).

The standard sperm analysis is the preferred and the most crowd-pleasing laboratory test in the diagnosis of male fertility. It evaluates sperm concentration, motility, morphology and viability. However, it is well known that normal results of sperm analysis cannot eliminate men from causes of couples’ infertility (Lewis *et al.,* 2008). Today, it is well known that the quality and integrity of sperm chromatin is very important in the reproductive capability of men because sperm DNA is known to contribute to half of the embryo’s genomic material. Our study showed that there was a significant increase in abnormal sperm chromatin in the cadmium-treated groups. In *in-vitro* systems, cadmium binds weakly to DNA (Valverde *et al.,* 2001), and there are many other cellular bio-ligands to which cadmium has high affinity to bind with, in particular the SH groups of thiols such as metallothioneins (Klaassen *et al.,* 1999). Therefore, the direct attack of DNA by cadmium may cause mutation. The most common cause of sperm DNA damage is oxidative stress (Barroso *et al.,* 2000; Kemal Duru *et al.,* 2000) and cadmium has the capacity to induce oxidative stress (Bal & Kasprzak, 2002; Nemoto *et al.,* 2009). In exposed cells and tissues, cadmium affects cellular thiol redox balance that leads to decreased intracellular glutathione content and reduced activities of cellular antioxidant enzymes (i.e. superoxide dismutase, peroxidase and catalase), which in turn results in the buildup of reactive oxygen species (ROS) and an increase in intracellular oxidative stress (Casalino *et al.,* 1997; Nemmiche *et al.,* 2011). The ROS might damage DNA through modification or deletions of bases, frame shifts, DNA cross-linkages, chromosomal rearrangement, single and double strand DNA breaks, and gene mutations (Aitken & Krausz, 2001; Spiropoulos *et al.,* 2002; Sharma *et al.,* 2004). Reactive oxygen species are produced in sperm through leakage of electrons from the mitochondrial electron transport chain (Vernet *et al.,* 2001), NADPH oxidase (Baker & Aitken, 2005) and generation of nitric oxide (Balercia *et al.,* 2004). The ameliorated DNA-damaged sperm by *C. lutea* extract might be due to its ability to maintain the level of zinc, an important regulator of DNA replication, transcription, and protein synthesis, influencing cell division and differentiation (Chia *et al.,* 2000).

Another factor, which is essential for spermatozoa metabolism and motility, is fructose, which serves as an energy source for spermatozoa. It is produced mainly by the seminal vesicles, with some contribution from the ampulla of the ductus deferens (Schoenfeld *et al.,* 1979). Determination of seminal fructose concentration has been used to examine obstructive azoospermia and inflammation of male accessory glands (Manivannan *et al.,* 2005). The result of this study showed that the seminal fructose level was significantly decreased in cadmium-treated rats. The diminution in seminal fructose level is in line with the Coppens (1997) report, which affirm that inflammation may lead to atrophy of the seminal vesicles and low seminal fructose concentration, and when ejaculatory ducts are blocked; fructose concentration in seminal plasma usually decreases and may become undetectable. Manivannan *et al.* (2005) also reported that fructose concentration in seminal plasma of patients with obstructive azoospermia is usually absent or significantly lower than that in men of normal fertility. Also, (Kise *et al.,* 2000; Kumar *et al.,* 2005) reported that the absence or reduced seminal fructose has been found in patients with congenital vas deferens-seminal vesicle developmental defect. On the other hand, there was significant increase in the seminal fructose level when cadmium was co-treated with *C. lutea* root methanol extract. The action of *C. lutea* methanol extract root may be attributed to its antioxidant properties.

In conclusion, the results of this study show that Cadmium has deleterious effects on male reproductive profile in experimental rat models, enough to cause infertility. *Carpolobia lutea* counteracts Cadmium-induced damage by ameliorating the toxic effects of Cadmium, thus enhancing reproductive activities.

## References

[r1] Agarwal A, Saleh RA, Bedaiwy MA (2003). Role of reactive oxygen species in the pathophysiology of human reproduction. Fertil Steril.

[r83] Aitken RJ, Clarkson J, Fisher S (1989). Generation of reactive oxygen species, lipid peroxidation and human sperm function. Biol Repro.

[r2] Aitken RJ, Harkiss DU, Buckingham DE (1993). Analysis of lipid peroxidation mechanism in human spermatozoa. Mol Repro Dev.

[r3] Aitken RJ, Krausz C (2001). Oxidative stress, DNA damage and the Y chromosome. Reproduction.

[r4] Ajibesin KK, Ekpo BA, Bala DN, Essien EE, Adesanya SA (2008). Ethnobotanical survey of Akwa Ibom State of Nigeria. J Ethnopharmacol.

[r5] Akinloye O, Arowojolu AO, Shittu OB, Anetor JI (2006). Cadmium toxicity: a possible cause of male infertility in Nigeria. Reprod Biol.

[r6] Al-Fartosy AJM, Sameerah A, Zearah SA, Alwan N (2013). Total antioxidant capacity and antihyperlipidemic activity of alkaloid extract from aerial part of Anethum graveolens L. plant. Eur Sci J.

[r7] Alvarez JG, Storey BT (1984). Assessment of cell damage caused by spontaneous lipid peroxidation in rabbit spermatozoa. Biol Reprod.

[r8] Baker MA, Aitken RJ (2005). Reactive oxygen species in spermatozoa: methods for monitoring and significance for the origins of genetic disease and infertility. Reprod Biol Endocrinol.

[r9] Bal W, Kasprzak KS (2002). Induction of oxidative DNA damage by carcinogenic metals. Toxicol Lett.

[r10] Balercia G, Moretti S, Vignini A, Magagnini M, Mantero F, Boscaro M, Ricciardo-Lamonica G, Mazzanti L (2004). Role of nitric oxide concentrations on human sperm motility. J Androl.

[r11] Ban Y, Asanabe U, Inagaki S, Sasaki M, Nakatsuka T, Matsumoto H (1999). Effects of alphachlorohydrin on rat sperm motions in relation to male reproductive functions. J Toxicol Sci.

[r12] Barroso G, Morshedi M Oehninger S (2000). Analysis of DNA fragmentation, plasma membrane translocation of phosphatidylserine and oxidative stress in human spermatozoa. Hum Reprod.

[r13] Benoff S, Hauser R, Marmar JL, Hurley IR, Napolitano B, Centola GM (2009). Cadmium concentrations in blood and seminal plasma: correlations with sperm number and motility in three male populations (infertility patients, artificial insemination donors, and unselected volunteers). Mol Med.

[r14] Casalino E, Sblano C, Landriscina C (1997). Enzyme activity alteration by cadmium administration to rats: the possibility of iron involvement in lipid peroxidation. Arch Biochem Biophys.

[r15] Chia SE, Ong CN, Chua LH, Ho LM, Tay SK (2000). Comparison of zinc concentrations in blood and seminal plasma and the various sperm parameters between fertile and infertile men. J Androl.

[r16] Cooper TG, Noonan E, von Eckardstein S, Auger J, Baker HW, Behre HM, Haugen TB, Kruger T, Wang C, Mbizvo MT, Vogelsong KM (2010). World Health Organization reference values for human semen characteristics. Hum Reprod Update.

[r17] Coppens L (1997). Diagnosis and treatment of obstructive seminal vesicle pathology. Acta Urol Belg.

[r18] Croute F, Beau B, Murat JC, Vincent C, Komatsu H, Obata F, Soleilhavoup JP (2005). Expression of stress-related genes in a cadmium-resistant A549 human cell line. J Toxicol Environ Health.

[r19] Duty SM, Calafat AM, Silva MJ, Brock JW, Ryan L, Chen Z, Overstreet J, Hauser R (2004). The relationship between environmental exposure to phthalates and computer aided sperm analysis motion parameters. J Androl.

[r20] Egbunike GN, Branscheid W, Pfisterer J, Holtz W (1986). Changes in porcine sperm lactate dehydrogenase isoenzymes during sperm maturation. Andrologia.

[r21] El-Demerdash FM, Yousef MI, Kedwany FS, Baghdadi HH (2004). Cadmium-induced changes in lipid peroxidation, blood hematology, biochemical parameters and semen quality of male rats: protective role of vitamin E and β-carotene. Food Chem Toxicol.

[r22] Elekofehinti OO, Adanlawo IG, Komolafe K, Ejelonu OC (2012). Saponins from Solanum anguivi fruits exhibit antioxidant potential in Wistar rats. Ann Biol Res.

[r23] El-Missiry MA, Shalaby F (2000). Role of beta-carotene in ameliorating the cadmium-induced oxidative stress in rat brain and testis. J Biochem Mol Toxicol.

[r24] Feng HL, Han YB, Hershlag A, Zheng LJ (2007). Impact of Ca2+ flux inhibitors on acrosome reaction of hamster spermatozoa. J Androl.

[r25] Fouad AA, Qureshi HA, Al-Sultan AI, Yacoubi MT, Ali AA (2009). Protective effect of hemin against Cadmium-induced testicular damage in rats. Toxicology.

[r26] Hirsch A (2003). ABC of subfertility: male subfertility. BMJ.

[r27] Iwu MM, Anyanwu BN (1982). Phytotherapeutic profile of Nigerian herbs. I: Anti-inflammatory and anti-arthritic agents. J Ethnopharmacol.

[r28] Kao SH, Chao HT, Chen HW, Hwang TI, Liao TL, Wei YH (2008). Increase in oxidative stress in human sperm with lower motility. Fertil Steril.

[r29] Kasinathan S, Veeraraghavan K, Ramakrishnan S (1987). Effect of cadmium on the spermatogenesis of Rana hexadactyla Lesson. Acta Morphol Hung.

[r30] Kato M, Makino S, Kimura H, Ota T, Furuhashi T, Nagamura Y (2001). Sperm motion analysis in rats treated with adriamycin and its applicability to male reproductive toxicity studies. J Toxicol Sci.

[r31] Kemal Duru N, Morshedi M, Oehninger S (2000). Effects of hydrogen peroxide on DNA and plasma membrane integrity of human spermatozoa. Fertil Steril.

[r32] Kise H, Nishioka J, Satoh K, Okuno T, Kawamura J, Suzuki K (2000). Measurement of protein C inhibitor in seminal plasma is useful for detecting agenesis of seminal vesicles or the vas deferens. J Androl.

[r33] Klaassen CD, Liu J, Choudhuri S (1999). Metallothionein: an intracellular protein to protect against cadmium toxicity. Annu Rev Pharmacol Toxicol.

[r34] Kumar R, Thulkar S, Kumar V, Jagannathan NR, Gupta NP (2005). Contribution of investigations to the diagnosis of bilateral vas aplasia. ANZ J Surg.

[r35] Kusakabe T, Nakajima K, Suzuki K, Nakazato K, Takada H, Satoh T, Oikawa M, Kobayashi K, Koyama H, Arakawa K, Nagamine T (2008). The changes of heavy metal and metallothionein distribution in testis induced by cadmium exposure. Biometals.

[r36] Lafuente A, Márquez N, Pérez-Lorenzo M, Pazo D, Esquifino AI (2001). Cadmium effects on hypothalamic-pituitary testicular axis in male rats. Exp Biol Med.

[r37] Lewis SE, Agbaje I, Alvarez J (2008). Sperm DNA tests as useful adjuncts to semen analysis. Syst Biol Reprod Med.

[r38] Li X, Wu X, Huang L (2009). Correlation between antioxidant activities and phenolic contents of radix Angelicae sinensis (Danggui). Molecules.

[r39] Lifeng T, Shoulin W, Junmin J, Xuezhao S, Yannan L, Qianli W, Longsheng C (2006). Effects of fenvalerate exposure on semen quality among occupational workers. Contraception.

[r40] Manivannan B, Bhande SS, Panneerdoss S, Sriram S, Lohiya NK (2005). Safety evaluation of long-term vas occlusion with styrene maleic anhydride and its non-invasive reversal on accessory reproductive organs in langurs. Asian J Androl.

[r41] Mitaine-Offer A, Miyamoto T, Khan IA, Delaude C, Lacaille-Dubois MA (2002). Three new triterpene saponins from two species of Carpolobia. J Nat Prod.

[r42] Muanya CA, Odukoya OA (2008). Lipid peroxidation as index of activity in aphrodisiac herbs. J Plant Sci.

[r43] Mukhopadhyay D, Varghese AC, Nandi P, Banerjee SK, Bhattacharyya AK (2010). CASA-based sperm kinematics of environmental risk factor-exposed human semen samples designated as normozoospermic in conventional analysis. Andrologia.

[r44] Nemmiche S, Chabane-Sari D, Kadri M, Guiraud P (2011). Cadmium chloride-induced oxidative stress and DNA damage in the human Jurkat T cell line is not linked to intracellular trace elements depletion. Toxicol In Vitro.

[r45] Nemoto K, Miyajimaa S, Hara S, Saigusa R, Yamada S, Ekimoto M, Degawaa M (2009). Cadmium-induced acute testicular toxicity. J Health Sci.

[r46] Nwafor PA, Bassey AL (2007). Evaluation of anti-diarrhoeal and anti-ulcerogenic potential of ethanol extract of Carpolobia lutea leaves in rodents. J Ethnopharmacol.

[r47] Odebiyi OO, Sofowora EA (1978). Phytochemical screening of Nigerian medicinal plants II. Lloydia.

[r48] Organization for Economic Cooperation and Development (OECD) (2001). OECD Guideline for Testing of Chemicals. 425. Statistical Programme Version: 1.0.

[r49] Park YS, Kim MK, Lee SH, Cho JW, Song IO, Seo JT (2011). Efficacy of testicular sperm chromatin condensation assay using aniline blue-eosin staining in the IVF-ET cycle. Clin Exp Reprod Med.

[r50] Pasqualotto FF, Sharma RK, Nelson DR, Thomas AJ, Agarwal A (2000). Relationship between oxidative stress, semen characteristics, and clinical diagnosis in men undergoing infertility investigation. Fertil Steril.

[r51] Publicover S, Harper CV, Barratt C (2007). [Ca2+]i signalling in sperm--making the most of what you've got. Nat Cell Biol.

[r52] Rajeshwar Y, Gupta M, Mazumder UK (2005). Antitumor activity and in vivo antioxidant status of Mucuna pruriens (Fabaceae) seeds against Ehrlich ascities carcinoma in Swiss albino mice. Iran J Pharmacol Ther.

[r53] Raji Y, Udoh US, Mewoyeka OO, Ononye FC, Bolarinwa AF (2003). Implication of reproductive endocrine malfunction in male antifertility efficacy of Azadirachta indica extract in rats. Afr J Med Sci.

[r54] Reijo R, Alagappan RK, Patrizio P, Page DC (1996). Severe oligozoospermia resulting from deletions of azoospermia factor gene on Y chromosome. Lancet.

[r55] Salama AF, El-Bahr SM (2007). Effect of Curcumin on Cadmium-Induced Oxidative Testicular Damage in Rats. J Med Res Inst.

[r56] Sarkar SD, Maiti R, Ghosh D (2006). Management of fluoride induced testicular disorders by calcium and vitamin-E co-administration in the albino rat. Reprod Toxicol.

[r57] Schoenfeld C, Amelar RD, Dubin L, Numeroff M (1979). Prolactin, fructose and zinc levels found in human seminal plasma. Fertil Steril.

[r58] Schuh K, Cartwright EJ, Jankevics E, Bundschu K, Liebermann J, Williams JC, Armesilla AL, Emerson M, Oceandy D, Knobeloch KP, Neyses L (2004). Plasma membrane Ca2+ ATPase is required for sperm motility and male fertility. J Biol Chem.

[r59] Sen Gupta R, Sen Gupta E, Dhakal BK, Thakur AR, Ahnn J (2004). Vitamin C and vitamin E protect the rat testes from cadmium-induced reactive oxygen species. Mol Cells.

[r60] Sharma RK, Said T, Agarwal A (2004). Sperm DNA damage and its clinical relevance in assessing reproductive outcome. Asian J Androl.

[r61] Sim KS, Nurestri AM, Norhanom AW (2010). Phenolic content and antioxidant activity of Pereskia grandifolia Haw. (Cactaceae) extracts. Pharmacogn Mag.

[r62] Siu ER, Mruk DD, Porto CS, Cheng CY (2009). Cadmium industry testicular injury. Toxicol Appl Pharmacol.

[r63] Soetan KO (2008). Pharmacological and other beneficial effects of anti-nutritional factors in plants-a review. Afr J Biotechnol.

[r64] Spiropoulos J, Turnbull DM, Chinnery PF (2002). Can mitochondrial DNA mutations cause sperm dysfunction?. Mol Hum Reprod.

[r65] Stohs SJ, Bagchi D, Hassoun E, Bagchi M (2001). Oxidative mechanisms in the toxicity of chromium and cadmium ions. J Environ Pathol Toxicol Oncol.

[r66] Suarez SS, Pacey AA (2006). Sperm transport in the female reproductive tract. Hum Reprod Update.

[r67] Toyoda Y, Chang MC (1974). Capacitation of epididymal spermatozoa in a medium with high K-Na ratio and cyclic AMP for the fertilization of rat eggs in vitro. J Reprod Fert.

[r68] Trease GE, Evans WC (1989). Trease and Evans' Pharmacognosy.

[r69] Valverde M, Trejo C, Rojas E (2001). Is the capacity of lead acetate and cadmium chloride to induce genotoxic damage due to direct DNA-metal interaction?. Mutagenesis.

[r70] Vernet P, Fulton N, Wallace C, Aitken RJ (2001). Analysis of reactive oxygen species generating systems in rat epididymal spermatozoa. Biol Reprod.

[r71] Verstegen J, Iguer-Ouada M, Onclin K (2002). Computer assisted semen analyzers in andrology research and veterinary practice. Theriogenology.

[r72] Waisberg M, Joseph P, Hale B, Beyersmann D (2003). Molecular and cellular mechanisms of cadmium carcinogenesis. Toxicology.

[r73] Wang Y, Fang J, Leonard SS, Rao KM (2004). Cadmium inhibits the electron transfer chain and induces reactive oxygen species. Free Radic Biol Med.

[r74] Wong A, Chuan SS, Patton WC, Jacobson JD, Corselli J, Chan PJ (2008). Addition of eosin to the aniline blue assay to enhance detection of immature sperm histones. Fertil Steril.

[r75] World Medical Association, American Physiological Society (2002). Guiding principles for research involving animals and human beings. Am J Physiol Regul Integr Comp Physiol.

[r76] Xu LC, Wang SY, Yang XF, Wang XR (2001). Effects of cadmium on rat sperm motility evaluated with computer assisted sperm analysis. Biomed Environ Sci.

[r77] Yakubu MT, Jimoh RO (2015). Carpolobia lutea roots restore sexual arousal and performance in paroxetine-induced sexually impaired male rats. Rev Int Androl.

[r78] Yesilli C, Mungan G, Seckiner I, Akduman B, Açikgöz S, Altan K, Mungan A (2005). Effect of varicocelectomy on sperm creatine kinase, HspA2 chaperone protein (creatine kinase-M type), LDH, LDH-X, and lipid peroxidation product levels in infertile men with varicocele. Urology.

[r79] Yoshida M, Kitani T, Takenaka A, Kudoh K, Katsuda SI, Taya K, Kurokawa Y, Maekawa A (2002). Lack of effects of oxonolic acid on spermatogenesis in young male adult and aged Wistar rats. Food Chem Toxicol.

[r80] Zahoor A, Muhammad SK, Mudassir AK, Amin H, Jamil R (2010). Seminal fructose in various classes of infertile patients. Pak J Physiol.

[r81] Zhang Y, Xie QX, Pan SP, Zhang CX, Xiao LJ, Peng YL (2005). Comparison of three methods for evaluating acrosome reaction in human spermatozoa. Zhonghua Nan Ke Xue.

[r82] Zhou Y, Zheng M, Shi Q, Zhang L, Zhen W, Chen W, Zhang Y (2008). An epididymis-specific secretory protein HongrES1 critically regulates sperm capacitation and male fertility. PLoS One.

